# Bilateral Sturge-Weber and Phakomatosis Pigmentovascularis with Glaucoma, an Overlap Syndrome

**DOI:** 10.1155/2015/106932

**Published:** 2015-05-06

**Authors:** Bharat Patil, Gautam Sinha, Bhagabat Nayak, Reetika Sharma, Sadhana Kumari, Tanuj Dada

**Affiliations:** ^1^Dr. Rajendra Prasad Centre for Ophthalmic Sciences, All India Institute of Medical Sciences, New Delhi 110029, India; ^2^Katihar Medical College, Katihar, Bihar 854105, India

## Abstract

*Aim*. To report a case of bilateral Sturge-Weber and Phakomatosis pigmentovascularis with secondary glaucoma in a child. *Method*. Case report. *Results*. A 4-year-old male child was referred to us for control of intraocular pressure (IOP). Sleeping IOP was 36 mm Hg in right eye and 28 mm Hg in the left eye. The sclera of both the eyes showed bluish black pigmentation—melanosis bulbi. Fundus examination of both eyes showed diffuse choroidal hemangiomas with glaucomatous cupping. Nevus flammeus was present on both sides of face along all the 3 divisions of trigeminal nerve with overlying hypertrophy of skin and on left forearm. Nevus fuscocaeruleus was present on upper trunk. All skin lesions were present since birth and were stationary in nature. CT scan of head revealed left-sided cerebral atrophy. Intraocular pressure was controlled after treatment with topical antiglaucoma medications. Pulsed Dye Laser has been advised by dermatologist for skin lesions. Patient has been advised for regular follow-up. *Conclusion*. The two overlapping dermatological disorders and their association with glaucoma are a rare entity. Management should be targeted both for dermatological and eye conditions.

## 1. Introduction 

Phakomatosis pigmentovascularis (PPV) is a rare, sporadic dermal disorder; nearly 200 cases have been reported up to date [1]. Phakomatosis cesioflammea is the most common type of PPV, a combination of cutaneous hemangioma and pigmentary nevus. SWS is known to have overlap combination with other congenital vascular disorders like Parkes Weber syndrome [2] and Klippel-Trénaunay syndrome (KTS) [3].

However, case reports of association of Sturge-Weber syndrome (SWS) with PPV are limited [4–8]. We are reporting a case of bilateral Sturge-Weber and Phakomatosis pigmentovascularis with secondary glaucoma in a four-year-old child being managed on topical antiglaucoma medications.

## 2. Case Presentation

A 4-year-old male child was referred to us for control of intraocular pressure (IOP). Trabeculectomy surgery was started outside in the right eye but was abandoned after conjunctival peritomy. On reviewing operative records the reason was found to be scleral thinning. Patient was not using any topical or systemic antiglaucoma medications. History was taken from parents and was reliable. The child was a known case of seizure disorder and was on prophylactic treatment with oral Phenytoin, since the age of seven months.

His best corrected visual acuity was 6/96 in the right eye RE and in 6/30 LE, on Cardiff acuity test. Left eye was red and congested; an exposed silk suture knot was noted over superior conjunctiva in the right eye, which was probably used to close peritomy. Sleeping IOP with Perkin's tonometer was 36 mm Hg (RE) and 28 mm Hg (LE).

**Figure 1:**
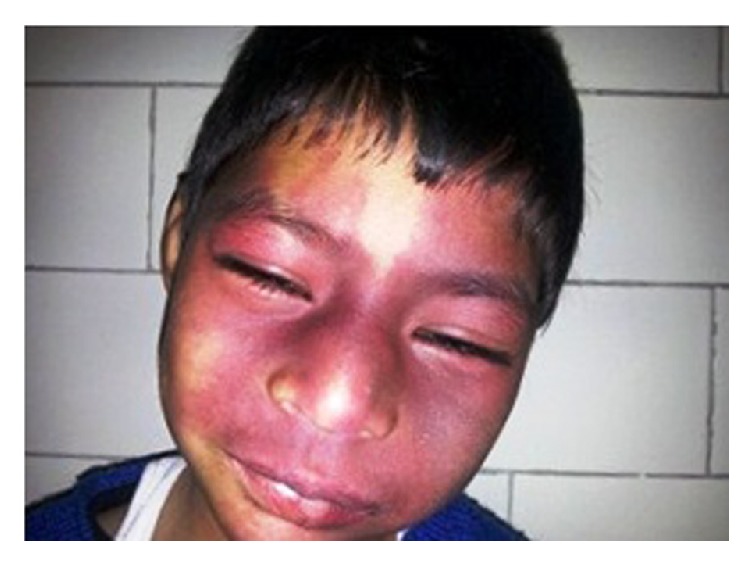
Bilateral port-wine stain over face.

**Figure 2:**
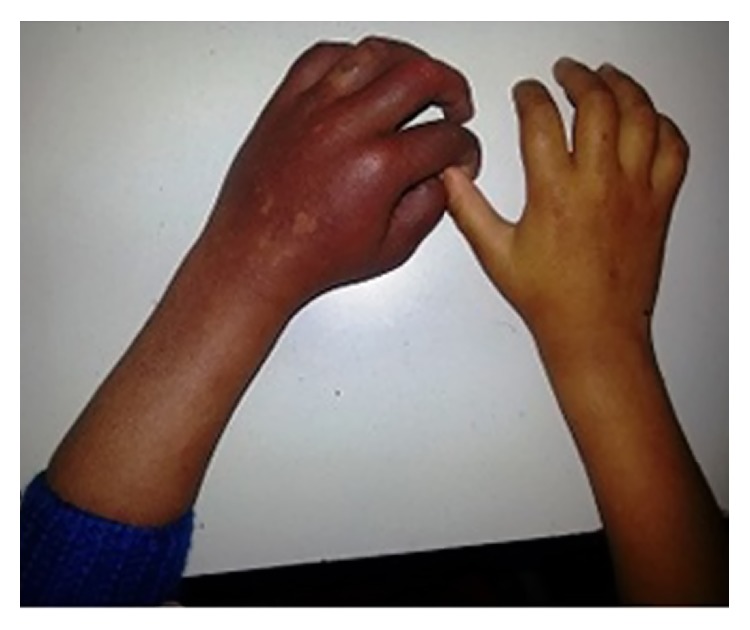
Port-wine stain of left forearm and disuse atrophy of right forearm and hand.

**Figure 3:**
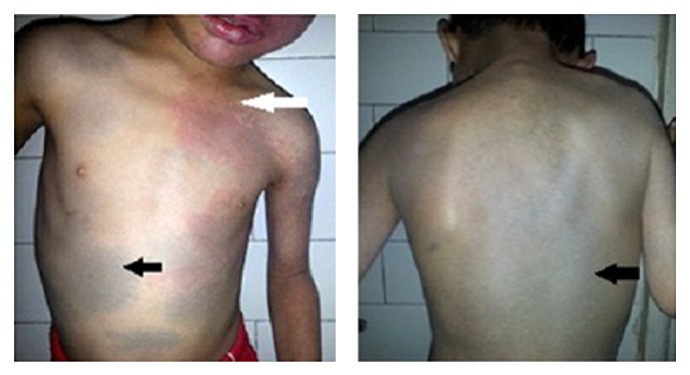
Mongolian spots (black arrow) and port-wine stain (white arrow).

**Figure 4:**
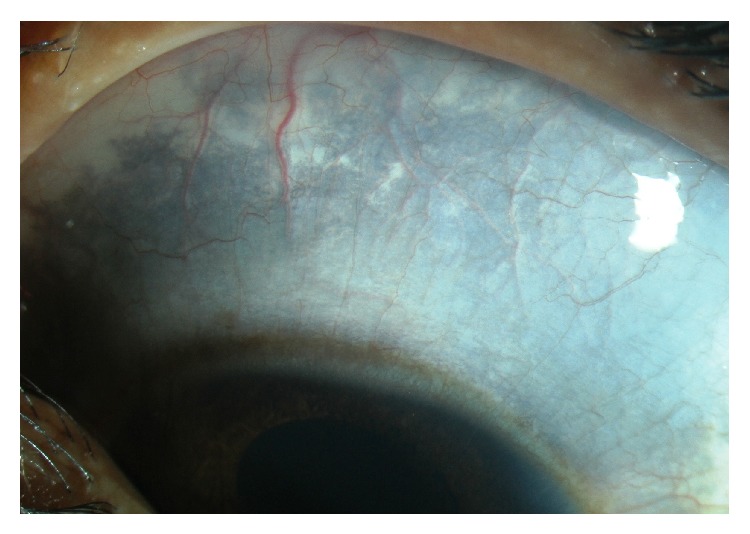
Ocular melanosis.

Systemic examination of the child showed the following skin lesions: (1) bilateral reddish pink macular lesion (nevus flammeus) involving face along all the 3 divisions of trigeminal nerve: V1, V2, and V3 distribution (Figure 1) with an overlying hypertrophy of skin. Similar lesion was present on left forearm (Figure 2); additionally, child had aberrant large, diffuse, well-defined blue-black asymptomatic macular dermal pigmentary lesions (nevus fuscocaeruleus) involving upper trunk (Figure 3). Thus diagnosis of Sturge-Weber syndrome along with Phakomatosis pigmentovascularis (PPV) was made. According to Happle's new classification PPV was classified as Phakomatosis cesioflammea (traditional types IIa and IIb). All lesions were present since birth and were stationary in nature. Oral mucosa was not having any vascular or pigmentary lesion. The sclera of both the eyes showed bluish black pigmentation—melanosis bulbi (Figure 4). No significant family history was elicited. No history of consanguinity was present.

Neuroimaging (CT scan of head) showed left-sided cerebral atrophy, without any intracranial calcification. There was no evidence of any other systemic vascular disorders. Clinical examination confirmed this finding and was suggestive of right upper limb and lower limb atrophy.

The child was started on eye drops Dorzolamide 2% three times a day, Timolol 0.5% two times a day, and Latanoprost (0.005%) at night time in both eyes. Examination under anesthesia done after 7 days of therapy showed IOP of 14 mm Hg in the right eye and 12 mm Hg in the left eye. Central corneal thickness on ultrasonic pachymetry was 595 *μ*m in the right eye and 605 *μ*m in the left eye. Corneal diameters were 13 mm × 12 mm in the RE and 12 mm × 12 mm in the LE. Axial length on A scan biometry was 22.5 mm and 22 mm in right and left eyes, respectively. Cup-disc ratio was 0.8 : 1 in the right eye with inferior notching and 0.6 : 1 in the left eye. Gonioscopy with Koeppe goniolens showed bilateral anterior and flat iris insertion. Fundus examination of both eyes with indirect ophthalmoscopy showed diffuse choroidal hemangiomas. Exposed silk suture knot was removed from the right eye, and peritomy revision with closure by 8-0 Vicryl suture was done. On follow-up at one month, the intraocular pressures were well controlled on the above regimen.

Dermatology consultation was done for management of port-wine stain, for which Pulsed Dye Laser was advised.

## 3. Discussion 

The Greek word “phaco” means “nevus.” Phakomatoses (or neurooculocutaneous syndromes, neurocutaneous disorders) are multisystem disorders that have characteristic central nervous system, ocular, and cutaneous lesions of variable severity. The skin and the brain have a common ectodermal origin, so there are many genetic and acquired diseases that affect both tissues. Phakomatosis pigmentovascularis is defined as an association of widespread vascular nevus with an extensive pigmentary nevus. There are two proposed theories for the pathogenesis of PPV. One theory proposes that PPV is a result of abnormalities in the development of vasomotor nerves and melanocytes. These cells derive from the neural crest, and their abnormal migration may lead to development of the nevus, while abnormal neural regulation may result in aberrant vascular development, leading to nevus flammeus or nevus anemicus. The association of dermal melanocytosis with cutaneous nevus flammeus is believed to result from a “twin spotting” phenomenon [9]. Sturge-Weber syndrome likely results from an early embryologic malformation of vascular development affecting the development of the nearby skin, eye, and brain structures. Studies suggest that complex molecular interactions contribute to the abnormal development and function of blood vessels in SWS [10]. Thus the vasomotor abnormality and twin spotting may explain the association of Sturge-Weber syndrome and PPV.

PPV was first defined in detail by Ota et al. in 1947 as a rare combination of cutaneous hemangioma and pigmentary nevus [1]. Since PPV is more common in Japan, the first case of association of Sturge-Weber syndrome and PPV is reported by Teekhasaenee and Ritch [4] in 1997; subsequently Hagiwara et al. [5] and Al Robaee et al. [6] reported similar cases. Gupta et al. in 2007 [7] reported a case of SWS in association with PPV and developmental glaucoma from India. Fernández-Guarino et al. [8] studied clinical findings of 15 patients with PPV and most frequent associations found were Sturge-Weber syndrome, Klippel-Trénaunay syndrome, and melanosis oculi. Sen et al. [11] reported a case with port-wine stain, Nevus of Ota, Sturge-Weber syndrome, and Klippel-Trénaunay syndrome.

Management of port-wine stain related glaucomas is controversial and difficult. These patients often have lower success rates as compared to other congenital glaucomas [12]. Cases associated with choroidal hemangioma have intraoperative risk of choroidal effusion up to 24% [13]. Oral propanolol has been used to reduce the incidence of choroidal effusion, but results are conflicting [14, 15].

Our case responded to medical management. It has been suggested that repetitive stretch and straining forces on the trabecular meshwork reduce the function of matrix metalloproteinases (MMP) and alter the extracellular matrix components, causing a further increase in IOP and that this may reduce the effective MMP levels exerted by Latanoprost [12]. Therefore, the presence of buphthalmos itself may reduce the effectiveness of latanoprost (0.005%). Further studies need to be conducted to confirm this hypothesis.

Our case is a rare association of Sturge-Weber syndrome, Phakomatosis pigmentovascularis, and developmental glaucoma. In view of increased incidence of intraoperative (expulsive hemorrhage) and postoperative (choroidal effusion) complications, initial treatment with topical drugs should be tried. Management should be targeted both for dermatological and eye conditions.
